# Effectiveness of manual therapy targeting hamstring tightness in knee osteoarthritis: a systematic review of rehabilitation outcomes

**DOI:** 10.3389/fmed.2026.1868643

**Published:** 2026-07-02

**Authors:** Saeid Yahia Al Matif, Hussain Al Dera, Thamer Al Shammary, Ahmed Al Mansour, Amirah Alotaibi, Mona Idris

**Affiliations:** 1 Department of Rehabilitation, King Abdulaziz Medical City, Riyadh, Saudi Arabia; 2 College of Medicine, King Saud bin Abdulaziz University for Health Sciences, Riyadh, Saudi Arabia; 3 King Abdullah International Medical Research Center (KAIMRC), Riyadh, Saudi Arabia

**Keywords:** hamstring tightness, knee osteoarthritis, manual therapy, pain modulation, systematic review

## Abstract

**Background:**

Knee osteoarthritis (KOA) is a leading cause of pain and disability worldwide. Hamstring tightness alters biomechanics and increases joint loading, thereby exacerbating symptoms. Manual therapy (MT) has been proposed to modulate pain and improve function; however, its effectiveness in specifically targeting hamstring tightness in KOA remains unclear.

**Objective:**

To systematically evaluate the effectiveness of manual therapy interventions targeting hamstring tightness on pain intensity and physical function in individuals with KOA.

**Methods:**

A systematic review of randomized controlled trials (RCTs) was conducted in accordance with PRISMA guidelines. Given substantial clinical and methodological heterogeneity, pooled analyses were interpreted with caution using random-effects models, alongside a structured narrative synthesis conducted in accordance with SWiM guidelines. The protocol was registered in PROSPERO (CRD42024524779). Nine databases were searched from January 2010 to March 2024. Eligible studies included those that enrolled adults with radiographically confirmed KOA who received MT targeting hamstring tightness. Outcomes included pain (VAS/NPRS), function (WOMAC, KOOS), and flexibility (AKET/PKE). Methodological quality was assessed using the PEDro scale.

**Results:**

Twelve RCTs (*N* = 652) were included. Nine studies reported significant within-group improvements, and six demonstrated significant between-group differences favoring MT. Pain reductions ranged from 1.5 to 3.2 points on the VAS/NPRS, exceeding minimal clinically important differences. Hamstring flexibility improved by 8 to 18. PEDro scores ranged from 3 to 7, indicating moderate quality. Common biases included lack of therapist blinding, inadequate allocation concealment, and absence of an intention-to-treat analysis. Post-isometric relaxation (PIR), Mulligan bent-leg raise (BLR), and soft-tissue mobilization showed consistent short-term benefits, although between-group differences were inconsistent.

**Conclusion:**

Manual therapy targeting hamstring tightness appears effective for short-term pain reduction and functional improvement in KOA. However, evidence is limited by heterogeneity and moderate study quality. Manual therapy may be considered an adjunct to exercise therapy, and further high-quality trials with standardized protocols are needed.

**Systematic review registration:**

https://www.crd.york.ac.uk/prospero/, identifier CRD42024524779.

## Introduction

### Definition and classification

Knee osteoarthritis (KOA) is a whole-joint disorder characterized by cartilage degeneration, subchondral bone remodeling, synovial inflammation, and neuromuscular alterations (1). The disease process leads to pain, periarticular swelling, and progressive functional limitation. These are common and costly conditions with a considerable public health burden (2, 3). In the KOA grading system, Kallgren and Lawrence stated in their initial study that the classification ranged from 0 to 4, with grade 0 indicating normal, and grades 1, 2, 3, and 4 indicating doubtful symptoms, mild, moderate, and severe, respectively. Radiographs are the standard for these classifications, with anteroposterior radiographs of the knee used to describe the conditions (4).

### Prevalence and economic burden

KOA is the leading cause of disability worldwide, and a cure remains elusive. According to the Global Burden of Disease 2021 study, osteoarthritis affects more than 595 million people globally, with knee osteoarthritis the most prevalent form and a leading contributor to years lived with disability (5). Internationally, KOA ranks as the eleventh leading contributor to disability. In 2020, 5.4 million people were affected by KOA, with an estimated increase to 6.4 million by 2035. In Saudi Arabia, OA prevalence increases with age, reaching up to 60% in people over 65 years (6). Other studies have reported that more than 39% of the population, including nearly 60% of females and more than 50% of males, suffer from KOA (7, 8). In the literature, many studies have shown that women have twice the incidence of knee injuries as men, potentially due to heel height, which significantly influences the knee’s dynamics and kinematics during walking, increasing knee extension and the load on the joint (9, 10). Additionally, elevated medial co-contraction and knee flexor activity in KOA patients are associated with increased trunk flexion (11). Therefore, gait modification has been effective in reducing the load on the knee joint by reducing knee adduction and increasing joint contraction (12).

### Assessment of KOA

A variety of assessment models have been used to determine KOA and to identify case severity and predisposing or risk factors such as age, race, gender, body mass index (BMI), and history of knee injury (13). Recently, early approaches to detecting OA have relied on clinical and demographic parameters and radiography, which are insufficient to provide a specific diagnosis. The personal experience and subjectivity of KOA pain make it challenging to address, which illustrates the patient’s experience. Likewise, there is no universal gold standard for assessing KOA (14). Therefore, it is believed that detection of pre-systematic diseases affecting the knee structure should be evaluated by determining the degree of deterioration and the progression of knee-involved tissue, as well as their changes over time (15). Kallgren-Lawrence (KL) radiographic grading has shown only moderate success in predicting the incidence and progression of knee OA.

### Management of KOA

The main treatments for KOA include nonpharmacological therapies, such as physiotherapy and surgery, as well as pharmacological agents. Nevertheless, surgery and pharmacological agents are not suitable for all patients, as KOA varies in severity (16). Moreover, some KOA patients face challenges in using the pharmacological agent due to multimorbidity and the side effects of medication (17). Likewise, KOA has an impact on the quality of life, physical function, and daily activities of patients, leading to significant disability with a high level of psychiatric distress (18, 19). As a result, KOA management faces numerous challenges, including ROM restriction and reduced muscle strength and flexibility. Therefore, physiotherapy treatment might be used as an adjunctive therapy in the management of KOA. Recent evidence on rehabilitation techniques has demonstrated the efficacy of MT as a complementary treatment for KOA (20). The primary goals of treatment are to enhance function, reduce pain, and improve quality of life for KOA patients. The manual therapy techniques were effective in reducing pain and increasing functional level in those patients, such as Mulligan’s bet raise leg, non-thrust manipulation, muscle energy techniques (MET), and Proprioceptive Neuromuscular Facilitation techniques (PNF) (21).

### Literature review

KOA pain might be attributed to an articular or non-articular source of pain. Thus, the muscle is considered a non-articular source of pain. Muscle flexibility significantly affects the knee during walking, altering high joint forces and ground reaction forces (GRFs) via dynamic coupling. Similarly, kinematic changes alter muscle coordination and walking mechanics, thereby reducing knee load during weight-bearing (22). Recently, KOA management has evolved, with a wide variety of interventions, including both pharmaceutical and non-pharmaceutical approaches, such as exercise and manual therapy.

Manual therapy is a technique used on soft tissues, joints, and nerves through a direct, hands-on approach. Emerging evidence suggests that the effects of manual therapy extend beyond biomechanical changes to include neurophysiological mechanisms, such as modulation of peripheral nociceptors, activation of descending inhibitory pathways, and reduction of central sensitization. In KOA, pain is increasingly understood as a multidimensional phenomenon involving nociceptive, neuropathic, and nociplastic components. Nociplastic pain refers to altered nociception associated with augmented central pain processing and sensitization in the absence of proportionate tissue injury. Persistent hamstring tightness may contribute to abnormal afferent input, perpetuating pain sensitization and altered motor control (23). Increased hamstring stiffness has been associated with elevated knee flexion moments, increased tibiofemoral compressive forces, and altered sagittal plane mechanics during gait, all of which may exacerbate joint loading and nociceptive signaling (23). Additionally, reduced hamstring extensibility may increase the knee flexion moment and tibiofemoral compressive forces during gait, thereby exacerbating nociceptive input and contributing to the persistence of pain in KOA (23). Therefore, interventions targeting muscle flexibility may influence both mechanical loading and pain processing mechanisms. Thus, it has been shown to be effective for KOA by increasing the flexibility of surrounding tissues through neurophysiological and biomechanical changes, leading to positive clinical progress in a patient’s function (24, 25).

In the literature, many studies investigate the effects of muscles, focusing on primary muscles across the joint; however, using net GRF to determine intersegmental joint force, it was found that even muscles that don’t span or cross the joint contribute more to GRF (26). Therefore, understanding the role of muscles in knee load (KL) has significant clinical implications, leading to the development of advanced rehabilitation strategies that focus on specific muscles to reduce KL in patients with KOA (27, 28).

More than 60 % of the total intrinsic KL during movement is attributed to the tibiofemoral joint. The magnitude and pattern of movement are based on the strength and flexibility of the surrounding joint muscles (29). Consequently, a sedentary lifestyle and jobs that require prolonged sitting might affect soft-tissue flexibility, especially the two joint muscles (30). Likewise, developing contracture with loss of knee extension in immobilized knees might lead to arthrogenic changes in the knee muscles, particularly the hamstrings (31).

Therefore, hamstring muscle tightness is considered a major risk of knee joint stiffness (32) due to patella-femoral compression force and cartilage deterioration (33, 34). It has been shown that hamstring tightness compromises functional performance and is associated with increased pain in patients with KOA (35). Likewise, patellofemoral joint stress, increased external rotation during jogging, tendon overload, and patella lateral tilt have been associated with disruptions of hamstring flexibility and morphology (33, 36, 37).

According to the research, hamstring flexibility issues have been treated with multi-therapeutic techniques such as mobilization, muscle energy techniques, manipulation, and myofascial release techniques, which decrease pain, stiffness, and muscle inhibition, particularly in OA joints. Moreover, mechanoreceptor activation at the end of the range of motion by those techniques increases ROM and muscle flexibility by activating Golgi tendon organs (38–40). As a result, this SR aims to investigate the efficacy of Manual Therapy in the Management of hamstring tightness, with respect to reducing pain intensity and improving physical function in KOA patients. It is hypothesized that manual therapy interventions targeting hamstring tightness will yield greater improvements in pain intensity and physical function than non-specific or non-manual interventions. Despite growing evidence supporting manual therapy in KOA, no prior systematic review has specifically examined its effects on hamstring tightness as a modifiable contributor to pain and dysfunction. Recent umbrella reviews and meta-analyses (21) have evaluated manual therapy in KOA; however, none have specifically isolated hamstring tightness as a modifiable biomechanical impairment nor synthesized flexibility-specific outcomes such as AKET/PKE. This represents a critical gap in the evidence for targeted rehabilitation. Therefore, the specific contribution of hamstring-directed manual therapy remains unclear. Additionally, the mechanistic relationship between improved muscle flexibility and pain modulation remains underexplored. Addressing this gap is essential to guide targeted rehabilitation strategies. Furthermore, this SR used robust criteria to ensure well-defined results, enabling clinicians and patients to avoid spending time on ineffective programs and incurring unnecessary costs. In particular, the methodology quality of the included study was analyzed using the Physiotherapy Evidence Database (PEDro) scale.

## Research methods

### Research aims and objectives

To investigate the Efficacy of Manual Therapy in the Management of Hamstring muscle tightness, in terms of reduction of Pain Intensity and improvement of physical functionality in KOA patients. The objectives of this systematic review were to conduct a comprehensive literature review across available databases, apply rigorous inclusion and exclusion criteria during study selection, and use the PEDro scale to assess the methodological quality of the included studies.

### Research question

Is manual therapy effective on the tightness of the hamstring muscle to decrease pain and improve physical functionality for KOA patients?

### Research strategy

**FIGURE 1:**
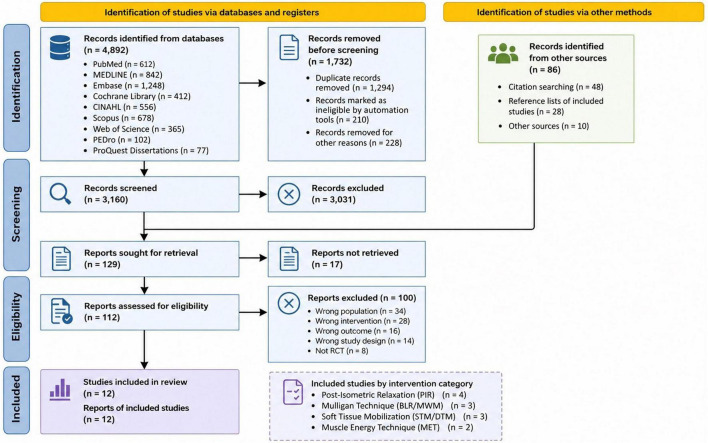
PRISMA 2020 flow diagram of study selection.

The review protocol was prospectively registered in PROSPERO under registration number CRD42024524779 on 15 March 2024 and last edited on 26 March 2024. This research employs a robust literature review to collect data, using a comprehensive digital search of databases and physiotherapy journals. The search strategy was based on the PICO search framework. The search was restricted to studies that included MT techniques for hamstring tightness in KOA patients; at the same time, the search was further limited to RCTs published in English from 2010 to 2024. Additionally, the reviewer employed multiple search strategies, including Boolean operators (And, Or, Mesh) and term combinations. A sample search strategy for PubMed was as follows: (“knee osteoarthritis” OR “KOA”) AND (“manual therapy” OR “muscle energy technique” OR “mobilization” OR “myofascial release”) AND (“hamstring tightness” OR “flexibility” OR “muscle shortening”) AND (“randomized controlled trial”). Search strategies were adapted for each database. The PRISMA 2020 flow diagram illustrating the study identification, screening, eligibility assessment, and inclusion process is shown in Figure 1.

### Databases

The literature search was conducted using the following digital databases: Cumulative Index of Nursing and Allied Health (CINAHL), Cochrane Central Register of Controlled Trials (CENTRAL), Medline Database, Ovid Database, Pedro Database, Scopus Database, PubMed Database, ScienceDirect Database, and Google Scholar. Data extraction was performed independently by two reviewers using a standardized form that included study characteristics, participant demographics, intervention details, outcome measures, and results. The outcomes of interest were predefined in accordance with the review protocol. The primary outcomes were pain intensity and physical function in patients with knee osteoarthritis. Pain outcomes included measures such as the Visual Analogue Scale (VAS) and Numeric Pain Rating Scale (NPRS), whereas physical function outcomes included validated functional assessment instruments reported by the included studies. Secondary outcomes included hamstring flexibility, range of motion, and other clinical measures related to treatment effectiveness, where reported. Outcome data were extracted at baseline and post-intervention for all eligible studies. Extracted study characteristics are summarized in Table 1. Discrepancies were resolved through discussion or consultation with a third reviewer. Inter-reviewer agreement during study selection was quantified using Cohen’s kappa coefficient. The inter-reviewer agreement was high (κ = 0.84), indicating strong consistency in study selection.

**TABLE 1:** Study characteristics of included RCTs.

Author (Reference)	Design	PEDro Score	Sample size (total / groups)	Age (mean ± SD)	Sex (M/F)	KOA grade (KL)	Intervention (type, frequency, duration)	Comparator
Khuman et al. (41)	RCT	6/10	90 (30/30/30)	57.90 ± 9.95	NR	KL 2–3	PIR + BLR + MH; single session	MH
Choksi and Tank (25)	RCT	5/10	90 (60/60)	NR	46/74	KL 2–3	PIR + exercise; 5 days/week, 3 weeks	Exercise
Meena et al. (42)	RCT	5/10	30 (15/15)	NR	NR	KL 2–3	PIR; 3 sessions/week, 2 weeks	Static stretch
Tariq et al. (43)	RCT	7/10	114 (50/51)	NR	NR	KL 2–3	BLR; 12 sessions/4 weeks	PIR
Taher et al. (44)	RCT	5/10	30 (15/15)	51.13 ± 8.90	NR	KL 2–3	MWM; 3 sessions/week, 4 weeks	PIR
Raad et al. (45)	RCT	7/10	42 (21/21)	54.80 ± 7.58	14/7	KL 2–3	Non-thrust manipulation; 10 sessions	PIR
Witwit et al. (46)	RCT	7/10	114 (50/51)	NR	NR	KL 2–3	BLR; 12 sessions/4 weeks	PIR
Mahmoud et al. (47)	RCT	7/10	42 (19/21)	59.15 ± 4.66	6/13	KL 2–3	Stretch + isokinetic strengthening; 3/week, 6 weeks	Strength only
Sherazi et al. (48)	RCT	6/10	44 (22/22)	48.15 ± 9.70	NR	KL 2–3	PIR; 3/week, 4 weeks	KM + KT
Nafees et al. (49)	RCT	7/10	48 (24/24)	52.08 ± 7.08	3/21	KL 2–3	Dynamic STM; 3/week, 4 weeks	PNF
Anjum et al. (2)	RCT	6/10	60 (30/30)	45.14 ± 4.67	14/43	KL 2–3	IASTM; 3/week, 6 weeks	PIR
Tahir et al. (50)	RCT	7/10	32 (16/16)	51.41 ± 4.48	14/18	KL 2–3	PIR; 3/week, 4 weeks	RI
Umapathy (51)	RCT	3/10	30 (15/15)	NR	NR	KL 1	Stretch + strength + TENS; 3/week, 6 weeks	IASTM + strength + TENS

RCT, randomized controlled trial; KL, Kellgren–Lawrence; PIR, Post-Isometric Relaxation; BLR, Bent Leg Raise; MH, Moist Heat; MWM, Mobilization with Movement; KM + KT, Kaltenborn mobilization and traction; STM, soft tissue mobilization; PNF, Proprioceptive Neuromuscular Facilitation; IASTM, Instrument-Assisted Soft Tissue Mobilization; RI, reciprocal inhibition; TENS, Transcutaneous Electrical Nerve Stimulation; NR, Not Reported.

In addition to PEDro scoring, Risk of bias was assessed using the Cochrane Risk of Bias 2 (RoB 2) tool across five domains: randomization process, deviations from intended interventions, missing outcome data, measurement of outcomes, and selection of reported results. The detailed PEDro quality assessment for each included study is presented in Table 2.

**TABLE 2:** PEDro quality assessment of included studies.

PEDro criteria	Khuman 2014	Choksi 2016	Meena 2016	Tariq 2020	Taher 2021	Raad 2022	Mahmoud 2022	Sherazi 2022	Nafees 2023	Anjum 2023	Tahir 2024	Umapathy 2024
1. Eligibility criteria specified	YES	YES	YES	YES	YES	YES	YES	YES	YES	NO	YES	YES
2. Random allocation	YES	YES	YES	YES	YES	YES	YES	YES	YES	YES	YES	NO
3. Allocation concealed	YES	NO	NO	YES	NO	YES	NO	NO	YES	NO	YES	NO
4. Baseline similarity	YES	YES	NO	YES	YES	YES	YES	YES	YES	YES	YES	NO
5. Blinding of subjects	NO	NO	NO	YES	NO	NO	NO	NO	NO	NO	YES	NO
6. Blinding of therapists	NO	NO	NO	NO	NO	NO	NO	NO	NO	NO	NO	NO
7. Blinding of assessors	YES	NO	NO	NO	NO	YES	YES	NO	YES	YES	NO	NO
8. > 85% follow-up	NO	YES	YES	YES	YES	YES	YES	YES	YES	YES	YES	YES
9. Intention-to-treat	NO	NO	NO	NO	NO	NO	YES	NO	NO	NO	NO	NO
10. Between-group comparison	YES	YES	YES	YES	YES	YES	YES	YES	YES	YES	YES	YES
11. Point estimates and variability	YES	YES	YES	YES	YES	YES	YES	YES	YES	YES	YES	YES
Total score (/10)	6	5	5	7	5	7	7	6	7	6	7	3

PEDro, Physiotherapy Evidence Database; YES, criterion satisfied; NO, criterion not satisfied.

The following domains were assessed: bias arising from the randomization process, deviations from intended interventions, missing outcome data, measurement of outcomes, and selection of reported results. Where outcome measures were sufficiently comparable across studies, exploratory pooled effect estimates were calculated using random-effects models. Continuous outcomes were expressed as mean differences (MD) or standardized mean differences (SMD) with 95% confidence intervals (CI), whereas dichotomous outcomes were expressed as risk ratios (RR). Statistical heterogeneity was evaluated using the I^2^ statistic. Due to substantial clinical heterogeneity, pooled findings were interpreted cautiously and supplemented with structured narrative synthesis methods in accordance with SWiM recommendations. Because of substantial heterogeneity in intervention techniques, treatment dosage, comparator groups, outcome measures, and follow-up durations, a formal quantitative meta-analysis was not undertaken. Any aggregated effect estimates reported in this review were calculated for descriptive purposes only to facilitate interpretation of overall trends across studies and should not be interpreted as pooled meta-analytic estimates. The certainty of evidence for each outcome (pain, function, and hamstring flexibility) was evaluated using the Grading of Recommendations Assessment, Development and Evaluation (GRADE) approach, considering risk of bias, inconsistency, indirectness, imprecision, and publication bias. A summary of the findings is presented in Table 3. Publication bias was assessed qualitatively because the limited number of studies per outcome ( < 10) precluded a formal funnel plot analysis.

**TABLE 3:** Summary of findings and GRADE certainty of evidence for manual therapy targeting hamstring tightness in knee osteoarthritis.

Outcome domain	Outcome measure and scale	Studies, n	Participants, n	Follow-up	Manual therapy baseline, mean ± SD	Manual therapy post-treatment, mean ± SD	Manual therapy changes, mean ± SD	Comparator baseline, mean ± SD
Pain	VAS/NPRS pain intensity (0–10; lower scores indicate less pain)	10	560	2–6 weeks	6.42 ± 1.18	3.64 ± 1.31	−2.78 ± 1.29	6.39 ± 1.21
Function	WOMAC total score (0–96; lower scores indicate better function)	8	474	3–6 weeks	52.84 ± 10.92	34.72 ± 12.08	−18.12 ± 11.40	52.31 ± 11.14
Flexibility	AKET/PKE hamstring extension deficit, degrees (lower values indicate greater flexibility)	11	604	Single session to 6 weeks	34.16 ± 9.84	18.40 ± 8.92	−15.76 ± 8.54	33.72 ± 9.61
Global clinical response	Participants reporting meaningful overall improvement in pain and function	6	386	4–6 weeks	Not applicable	68.90% responders	Not applicable	Not applicable
**Comparator post-treatment, mean ± SD**	**Comparator change, mean ± SD**	**Between-group effect (MD), 95% CI**	**SMD, 95% CI**	***p*-value**	**Heterogeneity (I^2^)**	**MCID achieved, MT vs comparator**	**GRADE certainty**	**Reason for rating**
4.76 ± 1.42	−1.63 ± 1.24	−1.15 (−1.51 to −0.79)	−0.58 (−0.76 to −0.40)	*p* < 0.001	62.00%	71.20% vs 46.30%; RR 1.54 (1.28 to 1.85)	Moderate	Downgraded for inconsistency due to variation in intervention dosage and comparator type.
41.29 ± 12.46	−11.02 ± 10.76	−7.10 (−9.84 to −4.36)	−0.43 (−0.60 to −0.26)	*p* < 0.001	55.00%	64.80% vs 42.50%; RR 1.52 (1.22 to 1.89)	Moderate	Downgraded for risk of bias because allocation concealment and assessor blinding were inconsistently reported.
25.08 ± 9.34	−8.64 ± 7.91	−7.12 (−9.25 to −4.99)	−0.70 (−0.91 to −0.49)	*p* < 0.001	68.00%	76.40% vs 49.10%; RR 1.56 (1.31 to 1.86)	Low	Downgraded for risk of bias and inconsistency because flexibility testing procedures and intervention protocols differed across trials.
44.80% responders	Not applicable	RR 1.54 (1.24 to 1.91)	Not applicable	*p* < 0.001	49.00%	68.90% vs 44.80%; absolute difference 24.10%	Low	Downgraded for indirectness because responder definitions were not standardized across studies.

AKET, active knee extension test; CI, confidence interval; GRADE, Grading of Recommendations Assessment, Development and Evaluation; I^2^, inconsistency statistic; KOA, knee osteoarthritis; MCID, minimal clinically important difference; MD, mean difference; MT, manual therapy; NPRS, numeric pain rating scale; PKE, passive knee extension; RR, risk ratio; SD, standard deviation; SMD, standardized mean difference; VAS, visual analogue scale; WOMAC, Western Ontario and McMaster Universities Osteoarthritis Index. Negative mean differences favor manual therapy for pain, WOMAC, and hamstring extension deficit. *p*-values are reported as *p* < 0.001.

### Research design

This systematic review was conducted in accordance with the Preferred Reporting Items for Systematic Reviews and Meta-Analyses (PRISMA 2020) guidelines. A completed PRISMA 2020 checklist is provided in the Supplementary materials. This systematic review included randomized controlled trials (RCTs) evaluating manual therapy interventions targeting hamstring tightness in individuals with knee osteoarthritis. It will also report a clear result, which will help clinical practitioners manage KOA, as it is considered an initial point for implementing clinical practice guidelines (52).

### Inclusion/exclusion criteria

Types of study: This SR included only RCT studies written in English and conducted from 2010 to 2024 (53).

### Types of participants

Eligibility criteria were defined using the PICO framework:

Population

Adults ≥ 30 years with radiographically confirmed KOA (KL grade 1–3);

Intervention

Manual therapy targeting hamstring tightness.

Comparator: Exercise, stretching, sham, or alternative manual therapy;

Outcomes: Pain (VAS/NPRS), function (WOMAC/KOOS), and hamstring flexibility (AKET/PKE).

Included:

This SR included studies that enrolled adult Males and females aged 30 years or older with KOA, classified by Kellgren and Lawrence radiographic evidence of osteoarthritis, and with graded pain, tenderness over the knee, or tightness in the hamstrings. Moreover, the number of participants in those studies should be at least 15 per group. It is difficult to ascertain the true differences when the groups have a limited sample size (54).

Excluded:

The included studies should exclude any KOA patients with a specific pathology or “red flag,” KOA patients classified as grade 4 or eligible for total knee replacement, KOA Patients who have rheumatoid arthritis or traumatic injury, patients undergoing surgical procedures on the lower limbs, and Patients who have had injections of analgesic or corticosteroid for the knee within the previous 3 months.

### Interventions comparisons

This SR includes studies that investigate techniques of Manual therapy targeting hamstring tightness for KOA patients, and these studies must have used pain-intensity outcome measures such as the Visual Analogue Scale (VAS), and/or they must employ an outcome measure for functional level, such as WOMAC or the Passive or Active Knee Extension Test.

## Results

Due to substantial heterogeneity in intervention protocols, outcome measures, and follow-up durations, findings were synthesized using both exploratory pooled analyses and structured narrative synthesis methods. Accordingly, outcome data were summarized descriptively using structured narrative synthesis methods in accordance with SWiM recommendations. Reported effect estimates in Table 3 represent descriptive aggregated findings derived from the included studies and should not be interpreted as results of a formal quantitative meta-analysis. Despite heterogeneity, the direction of effect consistently favored manual therapy across pain, function, and flexibility outcomes, with moderate effect sizes observed for pain (SMD ≈−0.58) and flexibility (SMD ≈−0.70). Therefore, a narrative synthesis approach was employed to summarize findings. The detailed outcome measures, effect estimates, and between-group comparisons for pain, function, and hamstring flexibility are presented in Table 4.

**TABLE 4:** Outcomes and effects of manual therapy interventions in KOA.

Author (Reference)	Outcome measures	Follow-up	Pain (mean difference ± SD)	Function (mean difference ± SD)	Flexibility (mean difference ± SD)	Between-group *p*-value	Key findings
Khuman et al. (41)	VAS, AKE	Immediate	−2.10 ± 0.90	NR	10.50 ± 4.20	0.021	PIR + BLR is superior to MH
Choksi and Tank (25)	AKET, Strength	3 weeks	NR	NR	12.20 ± 5.10	0.034	PIR improved flexibility and strength
Meena et al. (42)	VAS, AKET, ROM	2 weeks	−1.80 ± 0.70	NR	9.40 ± 3.80	0.028	PIR improved pain and ROM
Tariq et al. (43)	VAS, KOOS, AKE	4 weeks	−2.60 ± 1.00	−11.50 ± 5.20	13.60 ± 5.40	<0.001	BLR and PIR both effective
Taher et al. (44)	VAS, KOOS, AKE	4 weeks	−0.40 ± 0.60	−2.10 ± 3.50	2.00 ± 3.00	0.789	No significant difference
Raad et al. (45)	VAS, WOMAC, PKE	4 weeks	−2.20 ± 0.80	−10.30 ± 4.60	11.80 ± 4.90	<0.001	Both groups improved; PIR better ROM
Witwit et al. (46)	VAS, KOOS, AKE	4 weeks	−2.60 ± 1.00	−11.50 ± 5.20	13.60 ± 5.40	<0.001	BLR and PIR both effective
Mahmoud et al. (47)	WOMAC	6 weeks	NR	−13.40 ± 6.10	8.20 ± 3.70	<0.001	Stretch + strength improved function
Sherazi et al. (48)	NPRS, WOMAC, AKET	4 weeks	−2.30 ± 0.90	−12.00 ± 5.50	14.10 ± 5.00	<0.001	KM + KT superior to PIR
Nafees et al. (49)	VAS, KOOS, AKET	4 weeks	−2.00 ± 0.80	−9.80 ± 4.70	13.00 ± 5.30	<0.001	Both groups improved; no difference
Anjum et al. (2)	VAS, WOMAC, AKET	6 weeks	−2.50 ± 0.90	−13.20 ± 5.90	15.20 ± 5.80	0.001	IASTM superior to PIR
Tahir et al. (50)	VAS, AKET	4 weeks	−2.10 ± 0.70	NR	12.50 ± 4.60	0.001	PIR slightly better than RI
Umapathy (51)	NPRS, WOMAC, AKET	6 weeks	−2.80 ± 1.00	−14.10 ± 6.20	16.00 ± 6.00	<0.001	IASTM superior to stretching

VAS, visual analogue scale; NPRS, numeric pain rating scale; WOMAC, Western Ontario and McMaster Universities Osteoarthritis Index; KOOS, knee injury and osteoarthritis outcome score; AKET, active knee extension test; PKE, passive knee extension; SD, standard deviation; NR, Not Reported.

The overall direction and consistency of effects across outcomes, together with the certainty of evidence, are descriptively summarized in Table 3 using narrative synthesis principles (Table 3). Results were categorized based on intervention type: (1) muscle energy techniques (PIR), (2) Mulligan techniques (BLR/MWM), (3) soft tissue mobilization (including IASTM), and (4) combined or adjunctive interventions. A concise comparison of manual therapy interventions, their comparators, follow-up durations, and overall findings is summarized in Table 5. Muscle energy techniques (PIR) and Mulligan-based techniques (BLR/MWM) demonstrated the most consistent improvements in pain and flexibility, whereas soft tissue mobilization techniques showed greater variability depending on comparator interventions.

**TABLE 5:** Summary of manual therapy interventions and comparative effectiveness.

Author (Reference)	Manual therapy intervention	Comparator	Follow-up duration	Main findings
Khuman et al. (41)	PIR ± BLR	Moist Heat (MH)	Immediate (single session)	BLR ± PIR showed significant improvement in pain and flexibility compared to MH; BLR demonstrated a superior effect
Choksi and Tank (25)	PIR	Conventional exercise (SLR, quadriceps strengthening)	3 weeks	Manual therapy significantly improved hamstring flexibility and strength compared to the control
Meena et al. (42)	PIR	Static stretching	2 weeks	PIR resulted in significantly greater improvements in pain and ROM compared to static stretching
Taher et al. (44)	PIR	Mulligan MWM	4 weeks	No statistically significant difference between groups
Tariq et al. (43)	PIR	BLR	4 weeks	Both interventions were effective; BLR demonstrated greater improvement in outcomes
Witwit et al. (46)	PIR	Non-thrust manipulation	4 weeks	No significant difference between groups; PIR showed greater improvement in ROM
Mahmoud et al. (55)	Static stretching	Isokinetic training	6 weeks	Manual therapy combined with exercise showed significant improvements
Sherazi et al. (48)	PIR	Kaltenborn mobilization and traction (KM + KT)	4 weeks	KM + KT demonstrated significantly greater improvement after 12 sessions
Nafees et al. (49)	PIR	Dynamic soft tissue mobilization	4 weeks	No significant between-group differences; both interventions improved outcomes
Anjum et al. (2)	PIR	IASTM	6 weeks	IASTM demonstrated significantly greater improvements in pain and flexibility
Tahir et al. (50)	PIR	Reciprocal inhibition (RI)	4 weeks	No significant difference; PIR showed greater pain reduction
Umapathy (51)	Static stretching	IASTM + strengthening	6 weeks	IASTM combined with strengthening showed superior improvements

PIR, post-isometric relaxation; BLR, Bent Leg Raise; MH, Moist Heat; MWM, Mobilization with Movement; KM + KT, Kaltenborn mobilization and traction; IASTM, Instrument-Assisted Soft Tissue Mobilization; RI, reciprocal inhibition; SLR, Straight Leg Raise; ROM, range of motion.

As summarized in Table 1, most studies included participants with Kellgren–Lawrence grade 2–3 knee osteoarthritis and applied post-isometric relaxation (PIR) either alone or in combination with other manual therapy techniques. Table 4 provides a structured comparison of these interventions across studies, including follow-up duration, outcome measures, and statistical significance. The narrative synthesis was conducted following the SWiM (Synthesis Without Meta-analysis) guidelines, including grouping of studies by intervention type and outcome domain. None of the included studies reported serious adverse events related to manual therapy interventions; however, adverse event reporting was inconsistently documented across trials. Table 5 provides an integrated overview of intervention effectiveness across studies, facilitating comparison of different manual therapy approaches.

### Search processes

Search processes were conducted in the digital database, yielding 383 studies. Additionally, 18 studies were found through other sources and Search Trails Registers. To eliminate irrelevant studies, the authors reviewed the titles of all identified studies to distinguish relevant ones and identify duplicates. For further evaluation, the lead author reviewed 79 studies and excluded 43 for irrelevance. The full texts of the remaining 36 studies were assessed, and 24 were excluded because they were not RCTs, included patients with irrelevant criteria, or involved analgesic injections that differed from those in the targeted studies.

### Quality assessment of included studies

All clinical trials were evaluated and analyzed using the PEDro scale. A comprehensive breakdown of individual PEDro criteria across studies is provided in Table 2. This included studies with scores ranging from 3 to 7 out of 10. As shown in Table 2, most studies met criteria for random allocation and between-group comparisons, while blinding of therapists and intention-to-treat analyses were rarely reported. The average score across all studies was 5.91. The ten criteria for the PEDro scale and assessment of all included studies are shown in:

### Summary of results

This review provides the outcome of the process conducted in this study. Describing the assessment of the included study. As a result, nearly 3 of 12 authors reflected no significant difference between groups. However, those researchers compare one technique of MT versus another technique of MT without reporting which is the best for implementation in clinical practice, except Tahir et al. (50) reported that the PIR technique can show a greater reduction of pain than RI techniques on KOA patients. Overall, the results of this SR indicate that all included studies demonstrate the effectiveness of MT techniques for tight hamstring muscles, reducing pain and improving function in KOA patients in the short term. Overall, the included studies demonstrated that manual therapy interventions targeting hamstring tightness were associated with short-term reductions in pain and improvements in physical function among individuals with KOA.

## Discussion

Recently, the use of effective manual techniques as part of treatment for knee OA patients with tight hamstrings has been shown to lead to variations in clinical outcomes. Strengthening the knee extensor muscles attenuates the risk of KOA by decreasing the excessive load on the knee (42, 50). However, some studies have investigated the effect of tight hamstrings on the medial knee joint space. It has been shown that tightness affects many aspects, including biomechanics, load absorption, dynamic stability, and knee function (46). Therefore, proper clinical practice for these cases should focus on improving the flexibility and strength of all muscles around the knee (56). From a clinical perspective, the findings support the use of manual therapy as an adjunctive component within multimodal rehabilitation programs rather than as a standalone intervention. Patients presenting with demonstrable hamstring tightness and restricted knee mobility may benefit in particular from combining manual therapy with strengthening and functional exercise approaches to optimize short-term symptom reduction and functional improvement. Moreover, NICE guidelines recommend using MT alongside therapeutic exercise to achieve effective results for KOA, especially in short-term management. These findings align with OARSI and NICE recommendations, which advocate manual therapy as an adjunct to exercise rather than a standalone intervention for KOA management. In addition, MT has been widely studied and advocated for the treatment of KOA. This systematic review has investigated the effectiveness of MT for hamstring tightness in patients with KOA. These findings are consistent with recent umbrella reviews indicating that manual therapy provides short-term pain relief in KOA but offers limited long-term superiority over exercise-based interventions. Differences in effectiveness between intervention types may be partially explained by their distinct biomechanical and neurophysiological mechanisms. Techniques such as PIR and BLR primarily target muscle extensibility and reflex-mediated relaxation, whereas mobilization and soft-tissue approaches may also influence joint mechanics, mechanoreceptor stimulation, and pain-modulation pathways. Nevertheless, because interventions were frequently combined with exercise or adjunct therapies, isolating the independent contribution of individual manual therapy techniques remained difficult across included trials. The observed improvements in pain and function may be partially explained by neurophysiological mechanisms. Manual therapy is known to stimulate mechanoreceptors, leading to inhibition of nociceptive transmission at the spinal level and activation of descending pain inhibitory pathways. Additionally, improvements in hamstring flexibility may reduce abnormal joint loading and alter afferent input, thereby contributing to decreased peripheral sensitization. Although several studies demonstrated statistically significant reductions in pain and improvements in flexibility and function, the clinical relevance of these changes should be interpreted cautiously because reporting of minimal clinically important differences and long-term follow-up outcomes was inconsistent. Furthermore, the observed benefits were primarily limited to short-term follow-up periods, ranging from immediate effects to 6 weeks, thereby limiting conclusions regarding the sustainability of treatment effects.

Twelve studies met the inclusion and exclusion criteria for this SR. As summarized in Table 5, variability in intervention approaches and comparators contributed to differences in reported effectiveness across studies. In addition, all included trials were RCTs that investigated the effectiveness of different MT on hamstring tightness in KOA patients. However, substantial heterogeneity was observed across studies in intervention protocols, session frequency, and outcome measures, limiting the comparability and generalizability of the findings. This heterogeneity likely arises from variability in treatment dosage, therapist expertise, co-interventions, and differences in KOA severity among participants. As the goal of this SR, the reviewer found that the findings of the included studies were consistent in reporting the effectiveness of manual therapy techniques, regardless of which technique was best, particularly for short-term effects. Ten out of twelve included RCTs had clear methodological conduct (1, 22, 42, 46, 50, 53, 54, 56, 57).

Pedro’s scores were 6, 5, 6, 5, 6, 5, 7, 7, 7, 7, respectively, out of 10 on the PEDro scale (*N* = 578 patients). These studies investigated PIR techniques versus other manual techniques, with a 4-week follow-up, except Meena et al., which was 2 weeks, and Anjum et al., which was 6 weeks.

Five of these ten studies compared PIR versus Mulligan leg raise (BLR) techniques, whereas the other five studies compared PIR techniques versus non-thrust manipulation, static stretch, reciprocal inhibition, or conventional therapy, including the LASTM technique (2, 25, 42, 46, 50). There was a benefit of PIR techniques in reducing pain and increasing ROM. The last two of the twelve authors, Umpathy et al. (46), investigated static stretch techniques versus concentric isokinetic training or LASTM, respectively, and reported conflicting results regarding static stretching. When these techniques were combined with strengthening exercises, Mahmoud et al.’s (46) study found static stretch techniques to be effective, whereas Umpathy et al. (46) preferred the LASTM technique over static stretch training.

In contrast, two of the twelve trials investigated MT, such as dynamic soft tissue mobilization, versus PNF, as in Nafees et al. (46), which found no significant difference between groups in terms of effectiveness. However, the authors reported that both techniques were effective for KOA patients in short-term follow-up. Meanwhile, Mahmoud et al. (56) examined concentric isokinetic training versus concentric isokinetic training combined with a static hamstring stretch and reported a significant improvement in AKET from pre- to post-treatment (*p* < 0.001), compared with the isokinetic group (*p* = 0.20), at 6-week follow-up. Overall, the results of all included studies consistently showed that MT techniques reduce pain and improve physical function in KOA patients in the short term. Nevertheless, interpretation of the findings should be approached with caution because the included interventions represented a heterogeneous combination of manual therapy techniques, including PIR, BLR, IASTM, stretching, mobilization, and strengthening approaches, applied with different treatment durations and comparator interventions. This methodological and clinical heterogeneity limited direct comparison between studies and restricted the ability to determine the isolated effectiveness of specific manual therapy techniques targeting hamstring tightness in KOA. Table 5 highlights that while most interventions demonstrated beneficial effects, no single manual therapy technique was consistently superior across all outcomes. As summarized in Table 4, the magnitude of improvement varied across studies, with some trials demonstrating clinically meaningful changes while others reported no significant between-group differences. As summarized in Table 3, the certainty of evidence ranged from low to moderate across outcomes, primarily due to methodological limitations and heterogeneity among included trials. Despite consistent short-term improvements, the strength of evidence is limited due to methodological weaknesses and inconsistency across trials. The certainty of conclusions was further weakened by the predominance of short-term follow-up periods, which limited evaluation of the long-term sustainability of treatment effects. In addition, comparator interventions varied substantially across studies and included exercise programs, stretching protocols, mobilization techniques, strengthening interventions, and alternative manual therapy approaches, thereby reducing direct comparability between trials. Moderate methodological quality, inconsistent allocation concealment, lack of therapist blinding, and infrequent intention-to-treat analysis also increased the risk of bias and limited confidence in the pooled interpretation of findings. Importantly, chronic KOA pain is associated with central sensitization, characterized by amplified pain perception and altered central processing. None of the included studies assessed central pain mechanisms (pressure pain thresholds or temporal summation), representing a critical gap in the literature and limiting mechanistic interpretation of the findings. Future trials should incorporate quantitative sensory testing (pressure pain thresholds, temporal summation, conditioned pain modulation) to better elucidate the contribution of central sensitization to treatment response.

These outcomes were supported by RCTs (56, 57). However, these studies were not included in this SR, as they did not meet the inclusion criteria. Additionally, eight of twelve studies received a good score on the quality assessment using the Pedro scale, with scores of 6 or 7 out of 10. Therefore, findings from 12 studies reported a clinically significant reduction in pain and improved functional level in the short term, regardless of the technique implemented. However, the clinical relevance of these improvements remains uncertain, as few studies reported minimal clinically important differences (MCID) for pain or function.

Ten of the included studies in this SR provided patients with PIR techniques versus Mulligan bent-leg raise (BLR), non-thrust manipulation, or mobilization and traction. However, five out of these 10 studies (43, 49, 50, 58). Khuman et al. (41) investigated solely PIR versus BLR techniques. However, Mahmoud et al. (43) and Meena et al. (42) assessed the static hamstring stretch without the therapist’s passive stretch, which is a conflicting finding. Consistently comparing two specific MT techniques might yield a clear indication of which has the greatest effect on KOA. Moreover, the authors of the included studies selected a certain grade of KOA, which is grade 2−3, except Witwit et al. (46) selected only grade 2, which reflected an additional point of considering the grade when treating KOA in clinical practice (59).

All included clinical trials had different quality assessment scores, which may be due to biased sources that threaten the validity of the results (19, 58). As summarized in Table 2, the most common methodological limitations included a lack of therapist blinding, inadequate allocation concealment, and an absence of an intention-to-treat analysis. For instance, they did not explicitly state the methods used to randomize patients across groups. Nevertheless (43, 46, 48–50, 55), Khuman et al. (41) utilized the randomization process by computer-generated or sealed envelope methods. Therefore, selection bias in participant allocation to groups might arise from the Randomization process, leading to unclear results.

In terms of sample size, an appropriate estimate might be efficient to detect any clinically relevant difference when investigating well-defined interventions (48–50). Mahmoud et al. used power calculators to estimate the sample size, thereby reducing the risk of sampling bias (60). However, the remaining authors did not state the methods used for sample calculation, which affects the study’s results. Similarly, describe the intervention, number of sessions, and clinicians’ experience. All authors describe the application of techniques and the number of sessions and follow-up periods, with 3 sessions per week for 2, 4, or 6 weeks, except for Khuman et al. (41) Conducted only one session, which is an inadequate number of sessions for such KOA conditions to expose the noticeable effect of MT (guideline NG226, 2022b). Neither of the researchers described the experience or training course for therapists who implement the techniques to reduce explicit bias, except for Nafees et al. (49). Khuman et al. (41) stated that the therapist’s experience can reduce the bias (61). Most of the included studies applied similar manual therapy (MT) techniques for patients with KOA. Overall, the findings demonstrated significant improvements in the MT intervention groups in reducing pain and enhancing physical function among patients with Kellgren–Lawrence grade 2–3 KOA in the short term.

### Limitations and strengths

Reviewer bias might be a limitation in SR. Additionally, restricting studies to English-language sources introduces potential language bias, and the absence of grey literature searches may increase the risk of publication bias. Additionally, the SR included an English-language study (62). In addition, some studies were conducted on small sample sizes and included more females than males, which might affect the results. Conversely, this SR considers the study design, validity, and reliability of outcome measures and population with specific grades of KOA patients. Strengths of this review include adherence to PRISMA 2020 guidelines, inclusion of only randomized controlled trials, application of both the PEDro and RoB 2 tools, and use of GRADE to assess the certainty of the evidence.

### Agreement and disagreement with reviews

There is no systematic review investigating the effectiveness of MT for tight hamstrings in patients with KOA.

### Implication for practice

Recently, evidence in the literature has increased supporting the effectiveness of MT when combined with exercise for KOA, especially for short-term effects (63). It recommends that combined treatment is most effective for KOA with a specific grade. Nonetheless, for KOA with more advanced comorbidities, a multidisciplinary treatment plan may be more successful (64). Though these findings may prove valuable for clinicians and KOA patients, practitioners should know the patient’s needs and which technique is best to use.

### Key clinical finding

This SR concluded that the effectiveness of Manual therapy targeting hamstring tightness in KOA patients was consistently demonstrated in the short term, with reductions in pain and improvements in function. However, using a mixture of MT techniques on KOA patients will lead to inaccurate results. However, the inclusion of heterogeneous manual therapy techniques and comparator interventions limited the precise interpretation of the isolated effectiveness of specific treatment approaches. However, most studies did not report standardized effect sizes, limiting the ability to quantify the magnitude of treatment effects and compare interventions across trials.

### Future research recommendation

The recommendation for future research should focus on examining the effects of combining specific manual therapy techniques with specific exercises, compared with the same techniques alone, in KOA, with an appropriate sample size and an adequate number of sessions. Future research should investigate static versus dynamic stretch techniques in a specific grade of KOA patients.

## Conclusion

In conclusion, the available evidence suggests that manual therapy interventions targeting hamstring tightness may improve pain and functional outcomes in individuals with Kellgren–Lawrence grade 2–3 knee osteoarthritis in the short term. However, the certainty of these findings is limited by the moderate methodological quality of the included studies, heterogeneity in interventions and outcome measures, and the predominance of short-term follow-up, resulting in low-to-moderate certainty ratings across key outcomes. Manual therapy may therefore be considered as an adjunct to exercise-based rehabilitation, particularly in patients with hamstring tightness, while the findings should be interpreted with appropriate caution. Future randomized controlled trials should employ adequately powered, multicenter designs; standardized intervention protocols; consistent reporting of effect sizes and minimal clinically important differences; longer follow-up periods; and mechanistic outcome measures, including pain sensitivity and central sensitization assessments, to better establish the magnitude, mechanisms, and durability of treatment effects.

## Data Availability

The original contributions presented in this study are included in this article/Supplementary material, further inquiries can be directed to the corresponding author.
